# License to Regulate: Noncoding RNA Special Agents in Plant Meiosis and Reproduction

**DOI:** 10.3389/fpls.2021.662185

**Published:** 2021-08-20

**Authors:** Wojciech Dziegielewski, Piotr A. Ziolkowski

**Affiliations:** Laboratory of Genome Biology, Institute of Molecular Biology and Biotechnology, Adam Mickiewicz University, Poznan, Poland

**Keywords:** meiosis, noncoding RNA, RNA interference, RNA-dependent DNA methylation, small RNA, plants

## Abstract

The complexity of the subcellular processes that take place during meiosis requires a significant remodeling of cellular metabolism and dynamic changes in the organization of chromosomes and the cytoskeleton. Recently, investigations of meiotic transcriptomes have revealed additional noncoding RNA factors (ncRNAs) that directly or indirectly influence the course of meiosis. Plant meiosis is the point at which almost all known noncoding RNA-dependent regulatory pathways meet to influence diverse processes related to cell functioning and division. ncRNAs have been shown to prevent transposon reactivation, create germline-specific DNA methylation patterns, and affect the expression of meiosis-specific genes. They can also influence chromosome-level processes, including the stimulation of chromosome condensation, the definition of centromeric chromatin, and perhaps even the regulation of meiotic recombination. In many cases, our understanding of the mechanisms underlying these processes remains limited. In this review, we will examine how the different functions of each type of ncRNA have been adopted in plants, devoting attention to both well-studied examples and other possible functions about which we can only speculate for now. We will also briefly discuss the most important challenges in the investigation of ncRNAs in plant meiosis.

## Introduction

The basis of sexual reproduction is the fusion of two haploid cells called gametes to form a diploid cell that is the beginning of a new individual (Villeneuve and Hillers, 2001). For this to be possible, the genetic material from both parents has to be shuffled, and the chromosome number is reduced by half during the production of gametes or the spores that will later form gametes. Reduction of ploidy occurs during a type of cell division specific for all eukaryotes called meiosis (Villeneuve and Hillers, 2001). During meiosis, the cell undergoes profound functional and structural changes, including chromosome condensation, formation of programmed DNA double-strand breaks (DSBs), homologous chromosome pairing, and reciprocal exchanges of genetic information (crossovers) between paired chromosomes (Hunter, 2015; Mercier et al., 2015). At the same time, genetic information must be specially protected against damage that could occur, for example, as a result of the activity of transposable elements (TEs). Due to the uniqueness and complexity of meiosis, as well as its special importance for the preservation of genetic variability, it is very strictly controlled (Hunter, 2015; Mercier et al., 2015).

After initiation of the meiotic pathway, a massive transcription switch has been observed to activate in many eukaryotes, including yeasts, plants, and mammals (Primig et al., 2000; Zhou and Pawlowski, 2014; da Cruz et al., 2016). Although the number of genes expressed during meiosis differs among species, transcriptomic studies have revealed recurring patterns of coexpressed genes, which can be grouped into clusters associated with specific stages of meiosis. The early wave of meiosis-related genes is expressed during S phase and is connected with recombination and homologous chromosome pairing, while the mid- and late waves are expressed during metaphase and the onset of anaphase (Winter, 2012; Margolin et al., 2014; Flórez-Zapata et al., 2016). Recent RNA-seq studies and genetic approaches have revealed that a large number of different noncoding RNAs (ncRNAs) are involved in the onset and progression of meiosis, performing diverse molecular functions (Lardenois et al., 2011; Atkinson et al., 2018).

Noncoding RNAs can be classified as small RNAs (sRNAs; up to 30 nt), medium-size ncRNAs (mncRNAs; 31–200 nt), and long ncRNAs (lncRNAs; over 200 nt). This partly reflects their biogenesis and mode of action, especially with regard to sRNAs vs. lncRNAs. However, the division into sRNAs, mncRNAs, and lncRNAs is somewhat arbitrary, as the boundaries between the different classes are not strict. To our knowledge, there is no information on the specific role of mncRNAs in meiosis; therefore, this group of ncRNAs will not be included in this review. Both sRNAs and lncRNAs have been found in meiotic transcriptomes.

Small RNAs are associated with a number of gene and transposon silencing phenomena, which are collectively known as RNA interference (RNAi; Castel and Martienssen, 2013). RNAi has been described in many eukaryotes, including animals, plants, and yeast, although it has been lost in the *Saccharomyces cerevisiae* lineage (Drinnenberg et al., 2009). Two types of sRNA molecules are central for RNAi: microRNAs (miRNAs), which are derived from genome-encoded single-stranded RNAs, and small interfering RNAs (siRNAs), which originate from the processing of double-stranded RNA (dsRNA) precursors, usually transposable elements (Castel and Martienssen, 2013). In the context of meiosis, it is worth distinguishing one additional group of sRNAs, that is, phased small interfering RNAs (phasiRNAs). phasiRNAs are secondary siRNAs generated from miRNA target transcripts by their processing and controlled degradation. All sRNAs are single-stranded noncoding RNAs that function as gene/transposon repressors, which can operate *via* the cleavage of target transcripts, inhibition of mRNA translation, or repression of chromatin modification (Castel and Martienssen, 2013).

All types of sRNAs have been found to be highly expressed in plant generative tissues and especially important in plant sexual reproduction (Song et al., 2012). The role of long ncRNAs in meiosis is much less studied, but it seems important, especially for the organization of chromosomes and definition of centromere chromatin (Topp et al., 2004). In this review, we attempted to present the current knowledge and understanding of the role of noncoding RNA-dependent processes in plant meiosis, often referring to similarities with other eukaryotes. We did not aim to thoroughly analyze the biogenesis of particular types of ncRNAs or the mechanisms of their action, as the complexity of the pathways related to ncRNAs is beyond the scope of this review. Therefore, we decided to include only the most basic information on ncRNA biogenesis, which is necessary for understanding their function. Instead, we focused on discussing the research and experimental work addressing processes for which the influence of ncRNA on the initiation and course of meiosis is known or could be deduced.

## miRNAs Regulate Developmental Changes in Plants by Affecting the Expression of Transcription Factors

Plant miRNA-encoding genes are transcribed by Pol II into hairpin-structured pre-miRNAs, which are then sequentially processed by a Dicer-like endonuclease (DCL) to generate mature miRNAs. Next, miRNAs are loaded onto Argonaute (AGO) effector proteins and usually transported to the cytoplasm to regulate their target gene expression (Voinnet, 2009). miRNAs recognize transcripts of their target genes based on sequence complementarity and repress their expression by transcript cleavage, which dominates in plants, or translational repression, which is common in animals (Voinnet, 2009). This pathway of gene silencing is often referred to as posttranscriptional gene silencing (PTGS; Hamilton and Baulcombe, 1999). miRNAs frequently target transcription factors (TFs); for example, it is estimated that approximately 66% of experimentally verified targets in crops are TFs (Tang and Chu, 2017). Of the miRNA-targeted TFs, many are involved in the control of stage transition in plant development, and blocking miRNA expression often leads to visible developmental defects (Mallory and Vaucheret, 2006).

As miRNAs are relatively short (usually 21-nt), and some level of mismatch between the miRNA and transcript is allowed, a single miRNA is capable of targeting different members of a gene family. For example, a single miR167 eliminates transcripts of ARF6 and ARF8, two TFs essential for correct gynoecium and stamen development in *Arabidopsis thaliana* (Wu et al., 2006). miR172 regulates floral transition and flower development by repressing the *AP2* TF gene family in *A. thaliana* (Aukerman and Sakai, 2003). This function is evolutionarily conserved, as it has been reported even in distantly related species, including soybean, maize, rice, and barley (Tang and Chu, 2017). Similarly, miR159 controls floral development by negatively regulating *GAMYB* genes belonging to MYB transcription factors (Tsuji et al., 2006). The absence of miR159 results in the degeneration of the tapetum, which is required for anther post-meiotic development (Papini et al., 1999). Consequently, a lack of miR159 results in male sterility (Millar and Gubler, 2005). The miR159-*GAMYB* pathway is conserved in higher plants and is present in both monocots, such as rice and barley (Tsuji et al., 2006), and dicots, such as Arabidopsis (Palatnik et al., 2007) and strawberry (Csukasi et al., 2012).

### MicroRNAs in Regulation of Gene Expression in Meiosis

Yang et al. (2011) sequenced the Arabidopsis male meiocyte transcriptome and identified the expression of several miRNA genes. Among the miRNAs identified was miR163, which is known to target five SAM-dependent methyltransferase genes; indeed, the expression of these genes was not detected in meiocytes, suggesting that they are suppressed by miR163 (Yang et al., 2011). It should be noted that only immature miRNAs were able to be detected by the authors due to the method applied. A recent study focusing on sRNAs in Arabidopsis, soybean, and cucumber meiocyte transcriptomes provided a more comprehensive picture (Huang et al., 2020). Although miRNAs were less abundant in meiocytes than in leaves in all three species, 10 to 29% of the miRNAs showed preferential expression during meiosis. One of them was miR167, which is upregulated in meiocytes of all three species and, as previously mentioned, targets genes encoding ARF transcription factors (Huang et al., 2020).

It has also been shown that mutations in genes encoding proteins involved in miRNA biogenesis and function can affect the expression of genes important for meiosis initiation and meiotic recombination in *A. thaliana* (Oliver et al., 2017). Mutants of *DCL*, *HYL1*, *HEN1*, *HST*, and *AGO1* showed increased expression of *SPO11-1*, a topoisomerase responsible for meiotic DSB formation; *DMC1* and *RAD51*, recombinases involved in the strand invasion step; *MSH4*, a component of the Class I crossover pathway; or *MUS81*, a structure-specific endonuclease acting in the Class II crossover pathway, among others (Oliver et al., 2017). These results, however, must be interpreted with caution because the tested mutants do not participate exclusively in miRNA pathways and the research material consisted of flower buds and not pure meiocytes.

### Potential Roles of miRNAs in Chromosome Condensation

Control of chromosome condensation that occurs in meiosis may depend on miRNAs, and indirect evidence for this relationship has been described. In Arabidopsis, mutations in miRNA machinery genes, such as *DCL1*, *HYL1*, *HEN1*, *HST*, *AGO1*, and *AGO4*, have been found to impair the fertility and morphology of reproductive organs (Oliver et al., 2016, 2017). The numbers of viable pollen mother cells (PMCs) and megaspore mother cells (MMCs) were significantly reduced in all the investigated mutants compared to wild-type plants (Oliver et al., 2017). Interestingly, the sterility effect was stronger for PMCs than MMCs, which is consistent with observations in rice and maize showing the much greater importance of RNAi in male rather than female sporogenesis (Liu et al., 2020c). Apart from *hst*, partial chromatin decondensation was observed in chromosomes in pachytene in all mutants tested. This was not associated with an observable alteration in the histone modification pattern (H3K9me2, H3K4me2/3, and H3K27me3), although the limited resolution of cytology-based techniques does not allow exclusion of the possibility that such changes have occurred (Oliver et al., 2016, 2017). This may suggest that miRNAs regulate chromatin remodeling factors that influence DNA accessibility (Oliver et al., 2014, 2017; Pradillo and Santos, 2018).

In addition to the abovementioned functions, some miRNAs have been shown to play an indirect role in meiosis by triggering phasiRNA processing (see below).

## Small Interfering Rnas are Crucial for Transposon Silencing and High Meiotic Dna Methylation Levels and are Involved in the Control of Gene Expression in Meiosis

### Transposon Inactivation *via* Two siRNA-Dependent Pathways

siRNAs, typically 21 or 24-nt in length, are formed by cleaving longer dsRNAs that are produced from single-stranded RNAs by the action of RNA-dependent RNA polymerases (RDRs) or by annealing with *cis*-antisense RNAs (Castel and Martienssen, 2013). Like miRNAs, siRNAs can induce PTGS when loaded onto AGO proteins, resulting in transposon silencing. This group of siRNAs is usually called natural antisense transcript siRNAs (NAT-siRNAs; Axtell, 2013; Fei et al., 2013). In plant reproduction, this role of siRNAs has been extensively studied in relation to the male and female gametophytes of *A. thaliana* (Slotkin et al., 2009; Olmedo-Monfil et al., 2010). In response to the transcriptional reactivation of the subset of retrotransposons, siRNAs are produced in large quantities, preventing retrotransposition. There are indications that this phenomenon takes place in a cell-nonautonomous manner, and siRNAs are produced in one cell (central cell) and then transported to other cells (egg cells). Interestingly, the production of siRNAs and AGO9 is essential for the proper development of female gametophytes, determining cell fate in Arabidopsis ovules (Olmedo-Monfil et al., 2010).

In addition to PTGS, a related mechanism exists in plants by which RNA molecules direct the DNA methylation of their target DNA sequences, called RNA-dependent DNA methylation (RdDM; Matzke and Mosher, 2014; Erdmann and Picard, 2020). In RdDM, siRNAs, typically 24-nt long, guide DRM methyltransferases to specific target loci in the DNA. Since these siRNAs lead to the deposition of repressive chromatin marks, they are often referred to as heterochromatic siRNAs (Axtell, 2013; Fei et al., 2013). siRNAs are generally produced from transposable elements (TEs) and repeats and are transcribed by plant-specific RNA polymerase IV (Pol IV) with the second strand synthesized by RNA-dependent RNA polymerase 2 (RDR2), which is cleaved into mature siRNA by Dicer enzymes (Matzke et al., 2015). This mainly occurs as a consequence of extensive heterochromatin decondensation during male gametogenesis in vegetative cells and female gametogenesis in central cells (Slotkin et al., 2009; Ibarra et al., 2012). Thus, the major role of RdDM includes silencing new transposon insertions by DNA methylation and constant reinforcement of DNA methylation patterns in existing TEs to prevent their transposition (Matzke and Mosher, 2014; Erdmann and Picard, 2020). Examples of the role of RdDM in the regulation of development-related genes include especially floral transition (*FWA*; Soppe et al., 2000), imprinted expression in endosperm (Vu et al., 2013), seed dormancy (Iwasaki et al., 2019), and fruit ripening (Cheng et al., 2018). However, recent data show that RdDM targeting of both TEs and genes is also directly active during meiosis (see below).

### Effects of siRNA-Guided RdDM on DNA Methylation in Meiosis

**Figure 1:**
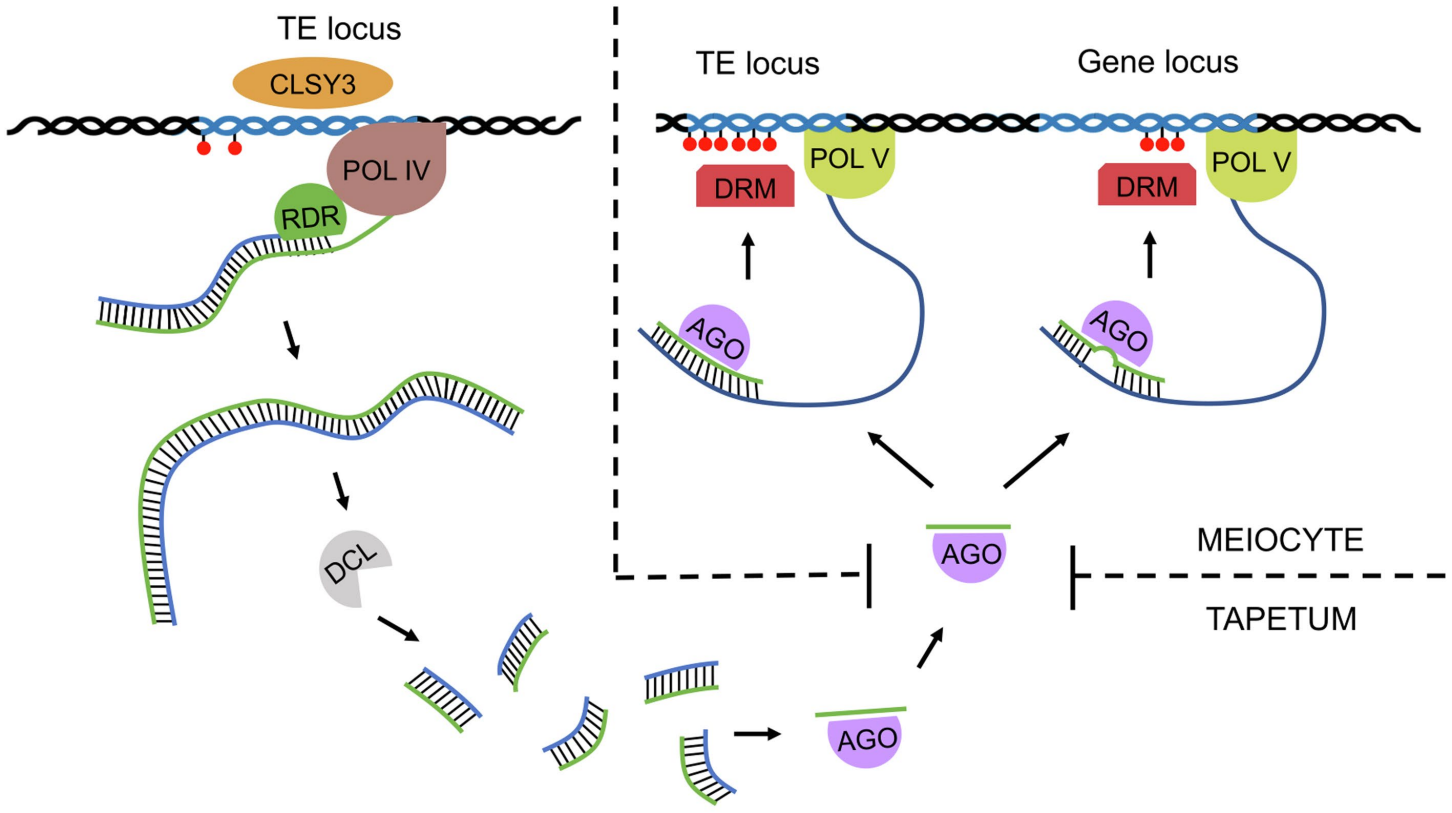
A simplified model of siRNA function in male Arabidopsis meiosis. The reproductive tissue-specific chromatin remodeler CLASSY3 (CLSY3) recruits Pol IV to some transposons in tapetum cells. The resulting transcripts are converted into double-stranded RNA (dsRNA) by RDR polymerase and then sliced by Dicer-like endonuclease (DCL) into 24-nt siRNAs. Single-stranded siRNAs are then loaded onto an Argonaute (AGO) and transported to meiocytes. There, siRNAs bind to nascent Pol V products, inducing local DNA methylation (red circles) *via* DRM methyltransferase. siRNAs perfectly match the transcripts of transposons from which they derive; however, in the case of genes, siRNAs usually pair imperfectly, allowing mismatches. Such gene methylation is germline specific, which indicates an increased sensitivity of RdDM machinery in meiocytes compared to somatic cells.

Reports based on whole-genome bisulfite sequencing have revealed changes in DNA methylation levels during sexual reproduction, which provides evidence for the extensive role of RdDM in Arabidopsis meiosis (Calarco et al., 2012; Walker et al., 2017). Unlike mammalian germ cells, the plant germline retains most DNA methylation, preventing transposon expression. It should be noted that in addition to canonical CG methylation, plants can also methylate DNA in the sequence contexts CHG and CHH, which does not occur in mammals. CHG methylation is also preserved in the plant germline, while asymmetric CHH methylation is lost from retrotransposons in meiocytes (Calarco et al., 2012; Walker et al., 2017). This CHH methylation is restored after fertilization by RdDM guided by 24-bp siRNAs (Jullien et al., 2012). Interestingly, nearly 1,300 loci were found to be hypermethylated in Arabidopsis meiocytes when compared to somatic tissues, and this DNA methylation was shown to be RdDM dependent (Walker et al., 2017). Hypermethylated loci occur not only in TEs (approximately 800 loci) but also in genes (nearly 500 loci; Long et al., 2021). In both cases, methylation is triggered by germline-specific 24-nt siRNAs, which are transcribed from TEs; however, genic methylation is established with imperfect sequence homology (Long et al., 2021). These siRNAs are produced in tapetum cells (nurse cells for PMCs; hence called nurse cell-derived siRNAs, niRNAs) with the help of the generative tissue-specific chromatin remodeler CLASSY3 (Zhou et al., 2021) and further transported to meiocytes *via* plasmodesmata (Figure 1; Long et al., 2021).

Most of the newly established genic methylation is germline-specific and results in corresponding gene repression in meiocytes (Walker et al., 2017). Sex lineage-specific hypermethylation has been observed for 84 pre-tRNA loci, the function of which remains unknown (Walker et al., 2017). One of the new targets is located within the last intron of *MPS1*, a gene involved in chromosome segregation during meiosis (Walker et al., 2017). Mutation in *MPS1* results in frequent occurrence of triads or polyads instead of tetrads (Jiang et al., 2009). In RdDM, the loss of DNA methylation in mutants disrupts the splicing of this intron, which creates a premature stop codon and, as a consequence, a nonfunctional protein (Walker et al., 2017).

The fact that tapetum-derived siRNAs arise from transposons and show only imperfect matches with genic sequences suggests that this type of regulation of meiotic gene expression evolved from the original RdDM task of preventing transposon expansion (Long et al., 2021). Although classical RdDM in somatic cells is sufficient to silence most transposons in Arabidopsis, some groups of transposons, e.g., those from the *Gypsy* family, are expressed specifically in the germline (Long et al., 2021). This explains why new targets of RdDM need to be established in meiocytes that establish germline lineages. However, it is not entirely clear why the RdDM pathway, which precisely targets DNA methylation in somatic cells based on a perfect siRNA target match, shows a relatively large number of off-targets in meiocytes. It is speculated that the activity of RNA polymerase V (Pol V), an important RdDM component, is elevated in meiosis, which can result in a higher number of off-targets (Long et al., 2021).

### Impact on Centromere Organization During Meiotic Division

Small RNAs, likely belonging to the siRNA class, play a role in the functioning of centromeres in plants (Gutbrod and Martienssen, 2020). Mutants of the rice meiosis-specific AGO gene *MEIOSIS ARRESTED AT LEPTOTENE1 (MEL1)* have been found to show impaired chromosome condensation in meiosis associated with major disruption of histone 3 lysine 9 methylation (H3K9me) at pericentromeres (Nonomura et al., 2007; Komiya et al., 2014; Fan et al., 2016). In addition, mutants of the AGO homologue AGO104 in maize have been found to show abnormal chromosome condensation, spindle defects, and aberrant chromosome segregation, leading to the presence of micronuclei in the later stages of meiosis (Singh et al., 2011). In both of these mutants, a disruption of centromeric and pericentromeric chromatin modification was observed, manifested by a significant decrease in DNA methylation in non-CG sequence contexts (Singh et al., 2011). The Arabidopsis AGO104 counterpart AGO4 also shows defects in chromosome segregation in mitosis and meiosis (Oliver et al., 2016). These effects are similar to the well-studied phenomenon of transcriptional gene silencing in fission yeasts: Transcription from repeated elements located in pericentromeric regions generates lncRNAs, which are then used to create siRNAs. siRNAs are loaded onto Ago1, which directs the RNA-induced transcriptional silencing complex (RITS) to the pericentromere through interaction with nascent lncRNAs. RITS in turn recruits the histone methyltransferase complex, which is responsible for H3K4me. The heterochromatin protein Swi6 binds to H3K9me and reinforces the heterochromatin state. Swi6 also participates in the recruitment of the cohesin complex, which holds sister chromatids together (Ellermeier et al., 2010; Gutbrod and Martienssen, 2020). While the exact process for forming centromeric heterochromatin with RNAi support is arguably different in plants than in yeasts, the functional similarities are striking together (Gutbrod and Martienssen, 2020).

## Secondary Sirnas Play Multiple and Diverse Roles in Male Meiosis

**Figure 2:**
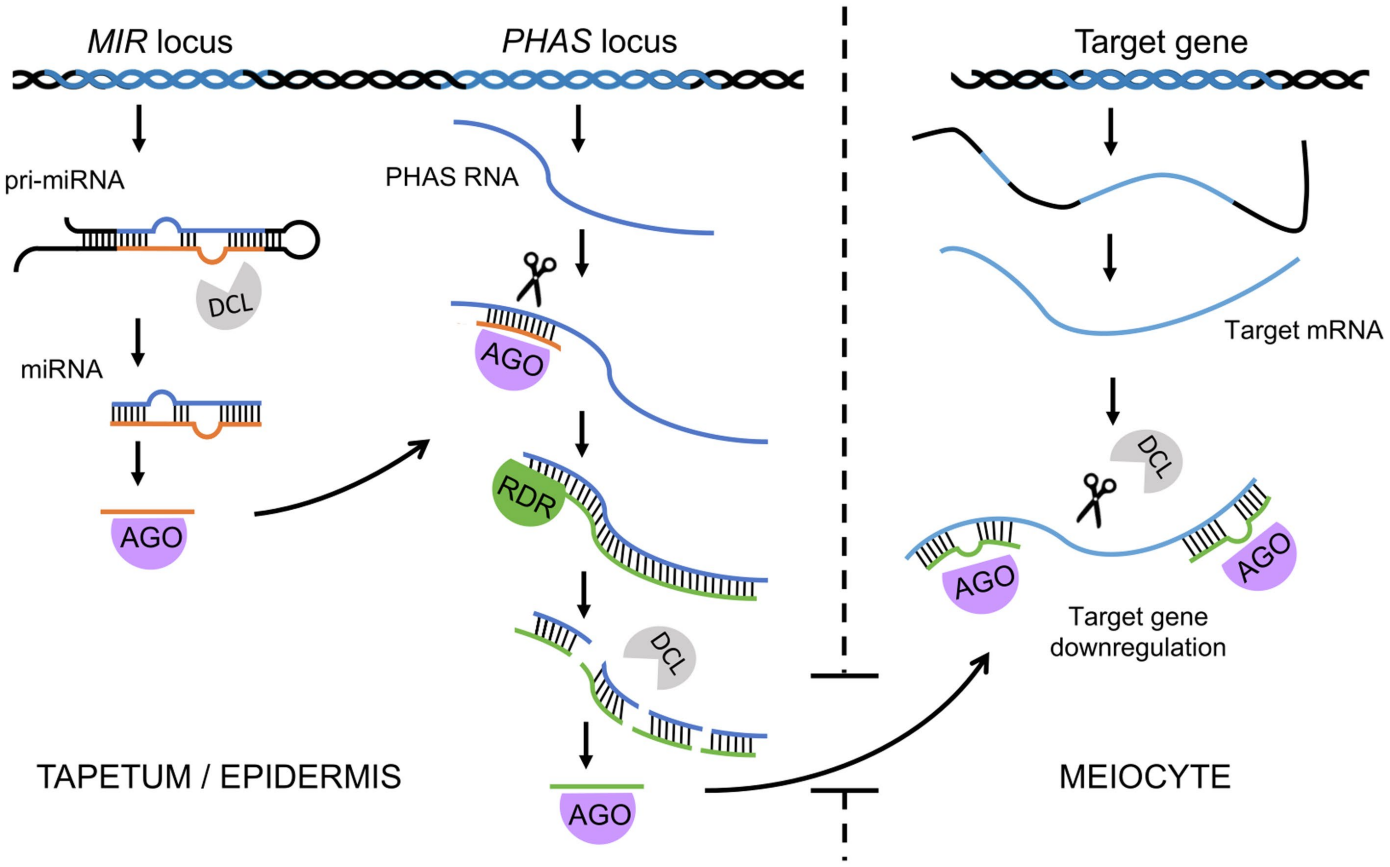
A simplified model for phased small interfering RNA biogenesis and function in male meiosis in grasses. miRNAs are transcribed from *MIR* loci, and after processing by Dicer (DCL), one miRNA strand is loaded onto the AGO protein. The miRNA-AGO complex binds to a *PHAS* transcript based on sequence complementarity, cleaves it at the binding site, and triggers synthesis of the second strand. dsRNA is then sliced by DCL starting from the cleavage site in a phased manner. The resulting phasiRNAs are then loaded onto another AGO protein (e.g., MEL1) and transported from the tapetum (21-nt phasiRNAs) or epidermis (24-nt phasiRNAs) to meiocytes. Finally, the phasiRNA-AGO complexes bind target transcripts of genes or transposable elements and, with the help of DCL, trigger their degradation.

In addition to primary siRNAs, whose precursor dsRNA is created by hybridization of independently transcribed RNAs, secondary siRNAs, whose precursor dsRNA synthesis is induced by an upstream sRNA trigger, also play an important role in plants (Axtell, 2013; Fei et al., 2013). PhasiRNAs are a recently discovered group of secondary siRNAs that seem to be of key importance in sporogenesis and male meiosis in numerous plants (Fei et al., 2013; Liu et al., 2020c). They are 21-nt or 24-nt secondary siRNAs generated by the processing of precursor transcripts triggered by miRNAs. Cleavage of the precursor transcript occurs at regular intervals; thus, phasiRNAs exhibit a distinctive, phased configuration (Figure 2; Johnson et al., 2009; Song et al., 2012). Reproductive phasiRNAs have been primarily identified in grasses but have more recently been confirmed to be widespread in flowering plants (Dukowic-Schulze et al., 2016; Kakrana et al., 2018; Huang et al., 2019; Yu et al., 2019; Huang et al., 2020). Recent work has reported the presence of reproductive phasiRNAs in dicots, although they are absent in legumes and Arabidopsis (Xia et al., 2019). In many plants, including Arabidopsis, rice, and maize, trans-acting siRNAs (tasiRNAs) have been described, whose name derives from their ability to target transcripts different than their source transcript, i.e., in *trans* (Vazquez et al., 2004; Allen et al., 2005; Adenot et al., 2006; Chen et al., 2010a). However, as many phasiRNAs can act in *trans* as well, tasiRNAs can be considered a subclass of phasiRNAs in which *trans*-targets have been identified (Axtell, 2013; Fei et al., 2013).

### 21-nt Reproductive phasiRNAs in Meiosis Progression

The 21-nt reproductive phasiRNAs are enriched mainly in early stage anthers and therefore are called premeiotic reproductive phasiRNAs (Zhai et al., 2015). This group of phasiRNAs is triggered by miR2118 (Johnson et al., 2009), and their production probably takes place in tapetum cells, from which phasiRNAs are transported to meiocytes (Zhai et al., 2015). Similar to other sRNAs, phasiRNAs function when loaded on AGO proteins. Research in rice and maize has revealed that 21-nt phasiRNAs in germ cells are bound by a specific AGO called MEL1 (known as AGO5c in maize; Nonomura et al., 2007; Komiya et al., 2014). Indeed, knockouts of *MEL1* show meiotic arrest in the early stages of prophase I and suggest that 21-nt premeiotic phasiRNAs directly or indirectly affect steps in meiotic progression, including chromosome compaction, presynaptic chromosome association, DSB formation, synapsis, and recombination (Nonomura et al., 2007; Komiya et al., 2014; Liu and Nonomura, 2016). Defects in chromatin condensation result from the limitation of prophase I histone modifications, in particular H3K9me2 (Nonomura et al., 2007; Liu and Nonomura, 2016). In centromeric regions, this leads to a partial mislocalization of centromere-specific histone 3 variant (Liu and Nonomura, 2016). It is currently unclear what causes the asynaptic phenotype observed in the *mel1* mutant. In this mutant, the distribution of PAIR2, a rice HORMA-domain protein, is no different from that in wild-type plants. On the other hand, the ZEP1 protein, a central transverse element of the rice synaptonemal complex, does not load properly onto chromosomes (Komiya et al., 2014). Rice shows recombination-dependent chromosome pairing, so one possibility is that the lack of synapses in the *mel1* mutant is a consequence of an almost complete absence of DSBs. Two additional AGO proteins, MAGO1 and MAGO2, have been recently identified in maize; these proteins are involved in the 21-nt reproductive phasiRNA pathway and accumulate mostly in anther epidermal cells and in developing meiocytes (Lee et al., 2020).

In rice, two loci encoding miR2118-dependent phasiRNA transcript precursors, *PMS1* and *PMS3*, have been isolated from a line used for hybrid rice production *via* photoperiod-induced male sterility (Ding et al., 2012a; Zhou et al., 2012; Fan et al., 2016). These loci were linked to DNA polymorphism that affects 21-nt phasiRNA production and results in male sterility (Fan et al., 2016). In the photoperiod-sensitive mutant line, male sterility is caused by premature degeneration of tapetum cells (Shi et al., 2009), suggesting that phasiRNAs affect the development of these cells. However, phasiRNA target identification is much more difficult than for other sRNA classes; targets therefore remain unknown in many cases. A recent study in rice revealed that 21-nt MEL1-dependent phasiRNAs bind and posttranscriptionally reduce the translation of nearly 2,300 proteins classified as adenyl ribonucleotide-binding proteins, kinases and hydrolases (Zhang et al., 2020a). These targets belong to genes rapidly downregulated in the transition steps observed during prophase I (Nelms and Walbot, 2019). Therefore, the function of 21-nt premeiotic phasiRNAs may be to eliminate the expression of specific gene categories at the onset of meiosis (Figure 2; Zhang et al., 2020a). On the other hand, a subclass of 21-nt miR2118-dependent phasiRNAs in maize was shown to be involved in male germline retrotransposon silencing under heat stress conditions (Lee et al., 2020). Inactivation of this pathway leads to male sterility at elevated temperatures, which is due to extensive transposon activation. This shows that 21-nt phasiRNAs may adopt different functions in male meiosis.

### Effects of 24-nt phasiRNAs on DNA Methylation

The 24-nt phasiRNAs are also highly enriched in anthers; however, at a later stage, coincident with meiosis, they are often referred to as meiotic phasiRNAs (Zhai et al., 2015). Their production is triggered mainly by miR2275 (Johnson et al., 2009), and they are likely to be loaded on the AGO18 effector protein (Fei et al., 2016). Several lines of evidence suggest that 24-nt phasiRNAs are produced in epidermis and tapetum cells and, to some extent, transported to meiocytes (Zhai et al., 2015; Dukowic-Schulze et al., 2016; Nan et al., 2017; Ono et al., 2018; Xia et al., 2019). Thus, as with Arabidopsis siRNAs and 21-nt phasiRNAs, 24-nt phasiRNAs must be delivered to meiocytes from nurse cells. Supplying the elements of individual RNAi systems from nurse cells appears to be a universal means of ncRNA-dependent meiocyte control (Lei and Liu, 2020).

Since the components of the phasiRNA biogenesis pathway are shared with other sRNAs, their mutants cannot be used to characterize the role of phasiRNAs in meiosis (Liu et al., 2020c). Fortunately for 24-nt phasiRNA research, the expression of their precursors in anthers depends on specific bHLH transcription factors, including rice ETERNAL TAPETUM1 (EAT1; Ono et al., 2018) and maize MS23 (Nan et al., 2017). Mutants of the genes encoding these TFs exhibit a male-sterile phenotype with delayed and asynchronous meiosis. Moreover, decondensed chromosomes were frequently observed in diakinesis and metaphase I in the *eat1* mutant (Ono et al., 2018). Mutation of DCL5, the Dicer protein responsible for miR2275 processing, has been found to result in almost complete loss of 24-nt phasiRNAs in maize (Teng et al., 2020). The maize *dcl5* mutants were male sterile, which was likely due to delayed development of the tapetum cells; the progression of meiosis was, however, not investigated in this contribution (Teng et al., 2020). Recent studies of both mutants in maize showed that 24-nt phasiRNAs can increase CHH methylation in most *PHAS* loci (Zhang et al., 2020b). As this latter study was performed in meiotic anthers and not in meiocytes, this DNA methylation profile is likely to correspond to abundant somatic tapetum cells (Zhang et al., 2020b). If we assume that in maize, as in Arabidopsis, the RdDM pathway is hyperactive in meiocytes (Long et al., 2021), it can be speculated that 24-nt phasiRNAs are capable of creating a germline-specific DNA methylation pattern in *trans*, providing a specific control for genes important in meiosis and additional silencing of transposable elements.

### Epigenetically Activated siRNAs in Triploid Block

Apart from phasiRNA, the plant germline contains another type of secondary siRNA – epigenetically activated siRNAs (easiRNAs; McCue et al., 2012; Creasey et al., 2014; Borges et al., 2018; Martinez et al., 2018). The formation of these siRNAs is related to the fact that in the germline, there is a significant decrease in DNA methylation in the CHH context, which in turn causes transient activation of retrotransposons (Creasey et al., 2014). easiRNAs arise from miRNA-dependent degradation of these retrotransposon transcripts shortly after meiosis (Borges et al., 2018). Interestingly, easiRNAs act as a quantitative signal for paternal genome dosage and are thus required for postfertilization genome stability and seed viability (Martinez et al., 2018). In the presence of easiRNAs, triploid seeds abort due to gene dysregulation, a phenomenon known as a triploid block. Depletion of easiRNAs bypasses the triploid block in response to elevated paternal ploidy, although it remains unclear how easiRNAs mediate this process (Borges et al., 2018; Martinez et al., 2018).

## Unclassified Meiocyte-Specific Small Rnas May be Associated with Meiotic Dsbs

Recent advances in the development of techniques for the isolation of pure PMC fractions in Arabidopsis have allowed for a more detailed analysis of sRNAs in meiosis. A group of approximately two thousand meiocyte-specific sRNAs (ms-sRNAs) of 23–24 nucleotides has been characterized based on comparison with the sRNAs present in leaves (Huang et al., 2019). Although ms-sRNAs require components of the RNAi machinery, including RNA Pol IV and DICER, for their formation, their origin remains unclear. Intriguingly, many of them map to genes that are upregulated in meiocytes and do not show meiosis-specific DNA methylation. Therefore, the authors hypothesized that this group of ms-sRNAs plays a role in the suppression of meiosis-specific genes in successive developmental stages (Huang et al., 2019). Even more interesting is the fact that approximately two-thirds of ms-sRNAs are not formed in the *spo11* mutant, which is defective for meiotic DSB formation, and this group is associated with common crossover hotspot motifs (CTT-repeat and A-rich) and open chromatin (Huang et al., 2019).

Recently, Liu et al. (2020a) characterized a new rice mutant allele for RNA-dependent RNA polymerase 6 (RDR6), which is necessary for dsRNA synthesis (Kumakura et al., 2009). The mutant showed a dramatic reduction in meiotic DSB formation, which resulted in the lack of chromosome pairing, lack of synapsis and pachytene arrest (Liu et al., 2020a). In this mutant, a significant increase in 24-nt sRNAs was reported, which is associated with a large number of downregulated genes, including some involved in DSB formation. Whether observations made by Huang et al. (2019) and Liu et al. (2020a) are related to each other currently remains unknown.

Although the function of these sRNAs in DSB repair needs to be further elucidated, they show similarities to qiRNAs in the filamentous fungus *Neurospora crassa* (Lee et al., 2009). qiRNAs arise in response to DNA damage in somatic cells and are also dependent on RNAi components, such as AGO and Dicer proteins as well as an RDR. Mutants of the genes involved in the production of qiRNA are sensitive to DNA damaging factors, suggesting that qiRNAs may be involved in DNA repair (Lee et al., 2009). In turn, in mammalian cells, a particular class of sRNAs is induced by DSBs (DSB-induced RNAs, diRNAs; Wei et al., 2012; Gao et al., 2014). In the Ago2 and Dicer mutants, which do not generate diRNAs, recruitment of RAD51 to DSBs is blocked. In contrast, proteins involved in DSB end processing, such as MRE11 and RPA, are normally loaded onto chromatin. This indicates the involvement of diRNAs in repair by homologous recombination (Gao et al., 2014).

## Long Ncrnas Provide Substrates for Srna Formation, Support the Formation of Centromeric Chromatin, and Can Potentially be Involved in the Regulation of Meiotic Gene Expression

Long ncRNAs are transcripts longer than 200 bp that are not translated into proteins (Chekanova, 2015). Similar to mRNAs, many are transcribed by RNA polymerase II and undergo similar processing, including 5'-end capping, splicing, and the addition of a polyA tail. In plants, however, there are two additional RNA polymerases that can lead to the formation of lncRNAs, i.e., polymerase IV and Pol V (Wierzbicki et al., 2008). As a rule, the transcripts of these polymerases do not undergo the usual mRNA processing and are therefore not stable. The resulting lncRNAs are usually substrates for the production of siRNAs involved in RdDM (Matzke and Mosher, 2014). The direct role of lncRNAs is widely known in yeast meiosis, where lncRNAs play a key role in the regulation of the meiotic switch and meiotic gene expression and in fission yeast even facilitate chromosome pairing (Van Werven et al., 2012; Ding et al., 2012b). Much less is known in plants than in yeasts about the importance of lncRNAs in meiosis. One of the reasons may be the very low expression level of long noncoding RNAs in plants, which makes them difficult to detect using the standard sequencing depth (Golicz et al., 2018).

### Potential Regulatory Roles of lncRNAs

The role of lncRNAs in developmental processes is well characterized in the context of floral transition. For instance, epigenetic regulation by three vernalization-dependent lncRNAs, *COLDAIR*, *COOLAIR*, and *COLDWRAP*, is important for inducing floral transition in *A. thaliana* by influencing the expression of *FLC*, the gene encoding a major repressor of flowering (Liu et al., 2010; Heo and Sung, 2011; Kim and Sung, 2017). Examples of lncRNAs acting in sexual reproduction are limited. One of them is *LDMAR* (long-day-specific male-fertility-associated RNA), which controls programmed cell death in developing anthers (Ding et al., 2012a). A certain amount of the *LDMAR* transcript is required for the normal development of pollen in rice grown under long-day conditions. Reduction of *LDMAR* transcripts leads to premature programmed cell death, resulting in photoperiod-sensitive male sterility (Ding et al., 2012a). Another example is *osa-eTM160*, which acts as an *miR160* sponge, an RNA containing complementary binding sites to miRNA. Expression of *osa-eTM160* at early anther developmental stages attenuates the repression of ARF mRNAs by *osa-miR160* and therefore regulates flower development (Wang et al., 2017).

Two recent studies have reported meiosis-specific lncRNAs isolated from plant meiocytes. Comparison of genes differentially expressed between sunflower meiocytes and somatic tissues allowed the identification of almost 7,000 lncRNAs that were exclusively expressed in meiocytes (Flórez-Zapata et al., 2016). At least 40% of these meiotically expressed lncRNAs showed sequence similarity with different sRNAs. In many cases, the identified lncRNAs are sRNA precursors or may mimic sRNA targets, thus acting like *osa-eTM160* as a miRNA sponge. Similarly, many of the nearly 5,000 lncRNAs specific for autotetraploid rice PMCs and MMCs were associated with miRNA and phasiRNA levels (Li et al., 2020). However, it seems very likely that some of these lncRNAs have additional regulatory functions, similar to those in yeast or in the regulation of developmental gene expression in Arabidopsis.

### Formation of the Centromeric Chromatin

In many eukaryotes, including plants, ncRNAs have been shown to influence the organization of chromatin in centromeres and appear to play a direct role in their function. Plant centromeres consist of the central core, containing satellite repeats, and the flanking pericentromeric heterochromatin. Centromeres have a dual function in cell division; they (1) enable the attachment of the CENH3 (equivalent to human CENP-A), which is required for kinetochore binding and karyokinetic spindle function, and (2) are essential for sister chromosome cohesion. In humans, loading of the CENP-A histone on the centromere has been shown to be dependent on the presence of centromere transcripts (Quénet and Dalal, 2014). Noncoding RNAs transcribed from human alpha satellites form complexes with CENP-A and CENP-C, and switching off their expression leads to the reduction of these proteins (McNulty et al., 2017). Similarly, the expression of centromeric retrotransposons and satellite repeats has been observed in maize (Topp et al., 2004). Nearly half of the resulting 40–200-nt noncoding RNAs are tightly bound to CENH3, suggesting that, as in humans, ncRNAs may play a key role in the formation of centromeric chromatin (Topp et al., 2004). More recent work indicates that among the ncRNAs linked to centromeric chromatin are also circular RNAs formed by back-splicing of some centromeric retrotransposons (Liu et al., 2020b). These circular RNAs bind to the centromere *via* RNA-DNA hybrids (the so-called R-loops) and give rise to chromatin loops and increase CENH3 accumulation. Although these observations were made based on the formation of centromeres in somatic cells, the convergent function of centromeres in mitosis and meiosis suggests at least partial universality of the processes.

The best-studied role of lncRNAs in plant meiosis is that of a substrate for the production of phasiRNAs. In this case, the lncRNA is transcribed from loci termed *PHAS* loci (*TAS* loci for tasiRNAs). Interestingly, not all *PHAS* loci are noncoding: A comprehensive study by Zheng et al. (2015) found that nearly half of the 3,300 *PHAS* loci identified in 23 plant species were protein coding. However, *PHAS* lncRNAs are only intermediates lacking biological functions separate from those of the phasiRNAs that form from them (Liu et al., 2020c).

## Concluding Remarks and Future Directions

**Figure 3:**
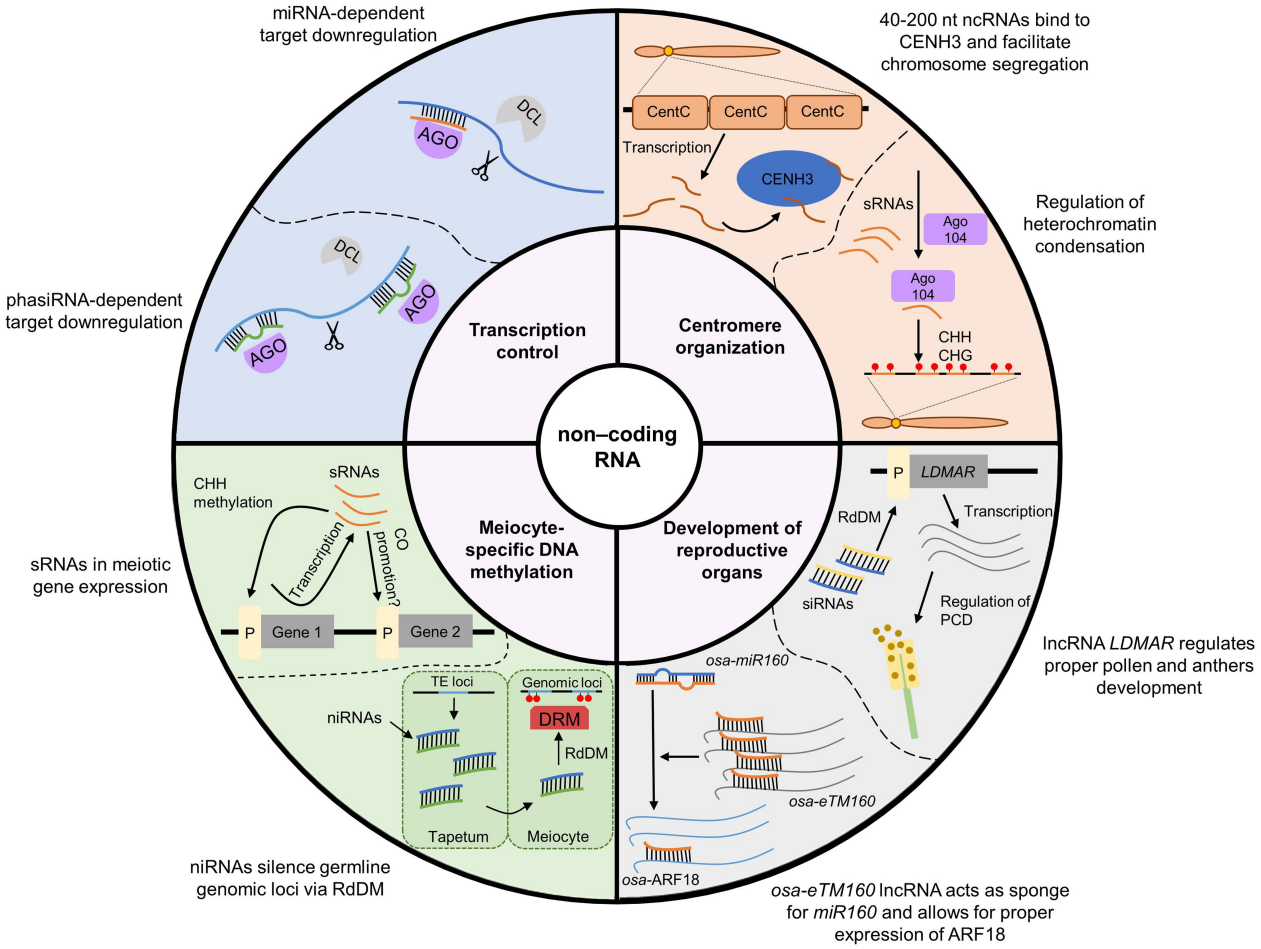
Summary of the most important functions of ncRNAs in meiosis in plants. For the sake of simplicity, only the most characteristic examples of individual processes are presented. A more detailed explanation is provided in Table 1.

The ncRNA-dependent mechanisms involved in the regulation of meiosis in plants are diverse and complex (Table 1 and Figure 3). This is mainly due to the existence of extensive and diverse regulatory pathways involving short RNAs, primary PTGS and RdDM, which are involved in RNAi (Figure 3). It is generally accepted that the primary function of the RNAi system is immune defense against exogenous genetic elements, such as transposons and viruses (Cerutti and Casas-Mollano, 2006). The role of RNAi in the regulation of gene expression, for example, through the miRNA pathway, appeared later in the course of evolution (Cerutti and Casas-Mollano, 2006). Since most functions of noncoding RNAs in meiosis can be classified as: (1) preventing mobile element activity, (2) regulating gene expression, and (3) controlling chromosome condensation, the high complexity of ncRNA-based mechanisms in plants may be due to the high level of transposons in their genomes. Consistent with this view, it appears that species with large genomes rich in transposable elements, such as maize, show a greater variety of ncRNA-based control mechanisms than do plants with small and simple genomes, such as Arabidopsis. One example of this is the reproductive tissue-specific phasiRNA, which is absolutely crucial for the fertility of many plants with complex, TE-rich genomes (Fei et al., 2013; Liu et al., 2020c).

**Table 1:** Role of ncRNAs in plant meiosis and reproduction.

Stage of sexual development	Type of ncRNA	Organism	Function	Described in:
Transcription control	miRNA	Flowering plants	miRNAs bind to targeted transcript and mediate its cleavage. Many different transcriptional factors, responsible for development of generative organs, are controlled by miRNAs. Moreover, some meiosis-specific genes are upregulated in *A. thaliana* mutants of miRNA machinery, suggesting their potential role in meiosis transcriptional control.	Tsuji et al., 2006; Oliver et al., 2017; Tang and Chu, 2017
	siRNA	Flowering plants	siRNAs prevent TE expansion in two pathways: (1) *via* PTGS by binding to TE transcripts to induce their cleavage and (2) *via* RdDM by guiding DRM methyltransferases to specific TE loci to establish CHG or CHH DNA methylation, which prevents new transposon insertions and transpositions. Moreover, siRNAs are responsible for restoring the CHH methylation after the fertilization.	Calarco et al., 2012; Walker et al., 2017; Erdmann and Picard, 2020
Meiotic double-strand break formation	SPO11-1-dependent sRNAs	*A*. *thaliana*	SPO11-1-dependent small RNAs are enriched with CO-associated DNA motifs and are related to CO hotspots and genes that lack DNA methylation. The exact function remains unknown.	Huang et al., 2019
Centromere organization	mncRNAs and lncRNA	*Z. mays*	mncRNAs and lncRNAs bind to CENH3 histone variant facilitating formation of centromeric chromatin, which is required for proper karyokinetic spindle function and for sister chromosome cohesion.	Topp et al., 2004
	siRNAs/phasiRNAs	*O. sativa*	siRNAs/phasiRNAs loaded onto MEL1 (rice) or AGO104 (maize) are responsible for RNA-mediated chromosome condensation in pericentromeres resulting from H3K9me and non-CG DNA methylation. Mutants of *MEL1/AGO104* exhibit meiocyte arrest, because of spindle defects and abnormal chromosome condensation.	Singh et al., 2011; Komiya et al., 2014
		*Z. mays*		
DNA methylation and silencing	meiocyte-specific sRNAs (ms-sRNAs)	*A*. *thaliana*	ms-sRNAs produced from meiotically expressed genes *via* RNA processing machinery are guided toward gene promoters to establish CHH methylation. High abundance of ms-sRNA is observed at Helitrons – a DNA transposon family known to overlap with DSB hotspots.	Huang et al., 2020
	siRNAs (niRNAs)	*A*. *thaliana*	niRNAs expressed in the tapetum from TE loci and subsequently transported to meiocytes trigger RdDM of transposons and genes.	Walker et al., 2017; Long et al., 2021
Floral transition	lncRNA	*A*. *thaliana*	Vernalization-dependent lncRNAs, *COLDAIR*, *COOLAIR*, and *COLDWRAP*, regulate the H3K27me3 levels by recruiting PRC2 at the *FLOWERING LOCUS C*, a major negative regulator of flowering.	Liu et al., 2010; Heo and Sung, 2011; Kim and Sung, 2017; Tian et al., 2019
Parental genome dosage	easiRNAs	*A*. *thaliana*	Pol IV is responsible for biogenesis of epigenetically activated siRNAs in the male gametophyte. easiRNAs mediate hybridization barriers between diploid seed and tetraploid pollen, by forming a quantitative signal for paternal chromosome number. easiRNAs target transcripts produced by Pol II or Pol V and interfere with DNA methylation establishment.	Borges et al., 2018; Martinez et al., 2018; Wang et al., 2020
Tapetum development	21-nt phasiRNAs	*O. sativa*	Expression of 21-nt phasiRNAs is triggered by miR2118 in the tapetum from *PHAS* loci. 21-nt phasiRNAs accumulate in anther epidermal cells and developing meiocytes, suggesting potential role of premeiotic phasiRNAs in the male meiosis initiation by affecting the expression of meiotic genes and contributing to retrotransposon silencing.	Johnson et al., 2009; Ding et al., 2012a; Zhou et al., 2012; Zhai et al., 2015; Lee et al., 2020; Fan et al., 2016
		*Z. mays*		
	24-nt phasiRNAs		Expression of 24-nt phasiRNAs is triggered by miR2275. Most likely, phasiRNAs are loaded on AGO18 effector protein and are responsible for proper tapetum development, by maintaining CHH DNA methylation levels.	Johnson et al., 2009; Zhai et al., 2015; Dukowic-Schulze et al., 2016; Fei et al., 2016; Xia et al., 2019; Zhang et al., 2020b
Anther development	lncRNA and siRNA	*O*. *sativa*	Long-day-specific male-fertility-associated RNA (*LDMAR*) is necessary for anther and pollen development. A specific siRNA named *Psi-LDMAR* has been associated with regulating levels of *LDMAR* transcript by targeting RdDM to its promoter region.	Ding et al., 2012a,b
	lncRNA and miRNA		A lncRNA *osa-eTM160* can mimic the target of osa-miR160 in order to release the repression of *osa*-*ARF18* gene, which is important in anther development.	Wang et al., 2017

TEs can integrate into genic sequences, rendering them inactive; therefore, they are dangerous to genome integrity (Ernst et al., 2017; Erdmann and Picard, 2020). Control of transposon activity is of particular importance in sexual lineages because new transposon insertions could be passed on to subsequent generations. Recent studies suggest that cells entering meiosis show increased activity of some RNAi components (Long et al., 2021), which may translate into increased off-target activity. Until now, it was widely accepted that mismatches are allowed in the case of sRNA acting in PTGS (Voinnet, 2009; Castel and Martienssen, 2013). However, recent studies have also shown that in the case of RdDM, a complete match between sRNA and the target is not required (Fei et al., 2021; Long et al., 2021). The biological significance of this fact is probably related to the rapid evolution of transposable elements: By allowing sRNA target mismatches in the PTGS and TGS RdDM pathways, it is possible to prevent the expansion of TEs that accumulate mutations rapidly. However, the consequence is a greater influence of sRNA-mediated processes on gene activity in meiosis. Hence, we propose that meiosis-specific regulation of gene expression through siRNA-related pathways (siRNA and phasiRNA) evolved as a side effect of the increasing activity and flexibility of these pathways in meiocytes in the fight against TE expansion.

In plants, we are just beginning to understand the importance of noncoding RNAs in meiosis. There are several reasons for such a situation. One of them is the parallel occurrence of many different ncRNA-mediated pathways, which can be difficult to differentiate. Especially, in the case of sRNAs, machinery required for sRNA biogenesis and functioning, including Dicer and AGO proteins, is often shared between individual pathways. It should also be taken into account that each pathway leads to the production of many different sRNAs having different targets. Therefore, the use of mutants for genes encoding components of these pathways is often not informative. Certain aspects of ncRNA functioning, however, can be solved using conditional mutants, where the expression of a given ncRNA synthesis component is limited to a selected tissue only. For example, Long et al. (2021) used an *rdr2* mutant into which they introduced a functional RDR2 under a tapetum-specific promoter. Thanks to this experiment, it was possible to establish that siRNAs directing DNA methylation in meiocytes are produced in tapetum cells and then transported to meiocytes.

Currently, the most frequently used approach is based on the identification of sRNAs by sequencing and their successive characterization. This is possible thanks to the rapid improvement in the efficiency of high-throughput sequencing techniques, often utilizing single-cell protocols and techniques for the isolation of super-pure meiocyte fractions (Chen et al., 2010b; Dukowic-Schulze et al., 2020). New techniques for isolating PMCs are based on microdissection (Chen and Retzel, 2013); however, in the case of grasses, flow cytometry is also successfully used, which significantly improves the throughput (Chang et al., 2018). It is, however, worth noting that while we have overcome the technical issues with PMC isolation in many plant species, we still do not have efficient methods for isolating MMCs. Therefore, in many cases, it is not possible to confirm to what extent the findings on male meiosis will be reflected in the formation of female gametophytes. In the genome-editing era, the development of new methods, such as CUT&RUN and CUT&Tag (Skene et al., 2018; Kaya-Okur et al., 2019), superresolution microscopy (Sydor et al., 2015), and systems enabling living-cell imaging for plant meiosis (Prusicki et al., 2019), should significantly expand our research capabilities.

## Author Contributions

WD and PAZ wrote the review. All authors contributed to the article and approved the submitted version.

## Conflict of Interest

The authors declare that the research was conducted in the absence of any commercial or financial relationships that could be construed as a potential conflict of interest.

## Publisher’s Note

All claims expressed in this article are solely those of the authors and do not necessarily represent those of their affiliated organizations, or those of the publisher, the editors and the reviewers. Any product that may be evaluated in this article, or claim that may be made by its manufacturer, is not guaranteed or endorsed by the publisher.
